# Bis[1-(3-cyano­benz­yl)pyridinium] bis­(1,2-dicyano­ethene-1,2-dithiol­ato)nickelate(II)

**DOI:** 10.1107/S1600536810051263

**Published:** 2010-12-11

**Authors:** Hua Xian, Hai-Bao Duan

**Affiliations:** a Department of Chemistry, Nanjing Xiaozhuang College, Nanjing 210017, People’s Republic of China

## Abstract

In the ionic title complex, (C_13_H_11_N_2_)_2_[Ni(C_4_N_2_S_2_)_2_], the Ni^II^ ion is located on an inversion centre so the asymmetric unit contains one-half [Ni(mnt)_2_]^2−^ dianion (mnt^2−^ is maleonitrile­dithiolate) and one 1-(3-cyano­benz­yl)pyridinium cation ([CNBzPy]^+^). The Ni^II^ ion in the [Ni(mnt)_2_]^2−^ anion is coordinated by four S atoms of two mnt^2−^ ligands, and exhibits square-planar coordination geometry. In the [CNBzPy]^+^ cation, the benzene and pyridine rings are twisted with respect to the C/C/N plane incorporating the methyl­ene C atom that links them. The crystal structure is stabilized by Coulombic inter­actions.

## Related literature

For background to the development of new functional mol­ecule-based materials, see: Robertson & Cronin (2002 ▶). For the applications of mol­ecular solids based on *M*[dithiol­ene]_2_ complexes in mol­ecular-based materials showing magnetic, superconducting and optical properties, see: Ni *et al.* (2004 ▶, 2005 ▶); Nishijo *et al.* (2000 ▶). For bond lengths and angles in related structures, see: Ren *et al.* (2004 ▶).
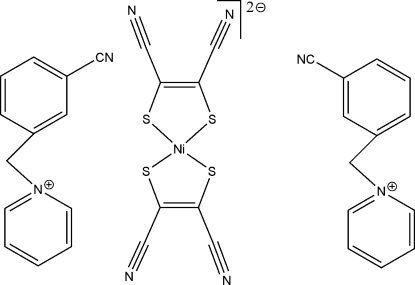


## Experimental

### 

#### Crystal data

(C_13_H_11_N_2_)_2_[Ni(C_4_N_2_S_2_)_2_]
*M* *_r_* = 729.57
Monoclinic, 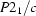

*a* = 11.633 (3) Å
*b* = 8.709 (2) Å
*c* = 16.692 (4) Å
β = 91.278 (5)°
*V* = 1690.7 (7) Å^3^
*Z* = 2
Mo *K*α radiation
μ = 0.86 mm^−1^
*T* = 293 K
0.4 × 0.3 × 0.2 mm

#### Data collection

Bruker SMART CCD area-detector diffractometer
Absorption correction: multi-scan (*SADABS*; Bruker, 2000 ▶) *T*_min_ = 0.702, *T*_max_ = 0.741
13953 measured reflections
3068 independent reflections
2154 reflections with *I* > 2σ(*I*)
*R*_int_ = 0.092

#### Refinement

*R*[*F* ^2^ > 2σ(*F* ^2^)] = 0.076
*wR*(*F* ^2^) = 0.130
*S* = 1.18
3068 reflections
214 parameters
H-atom parameters constrained
Δρ_max_ = 0.32 e Å^−3^
Δρ_min_ = −0.31 e Å^−3^

### 

Data collection: *SMART* (Bruker, 2000 ▶); cell refinement: *SAINT* (Bruker, 2000 ▶); data reduction: *SAINT*; program(s) used to solve structure: *SHELXTL* (Sheldrick, 2008 ▶); program(s) used to refine structure: *SHELXTL*; molecular graphics: *SHELXTL*; software used to prepare material for publication: *SHELXTL*.

## Supplementary Material

Crystal structure: contains datablocks global, I. DOI: 10.1107/S1600536810051263/bx2332sup1.cif

Structure factors: contains datablocks I. DOI: 10.1107/S1600536810051263/bx2332Isup2.hkl

Additional supplementary materials:  crystallographic information; 3D view; checkCIF report

## Figures and Tables

**Table:** 

Ni1—S1	2.1710 (13)
Ni1—S2	2.1713 (14)

**Table:** 

S1—Ni1—S2	87.64 (5)
S1^i^—Ni1—S2	92.36 (5)

Symmetry code: (i) 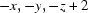.

**Fig. 1.**
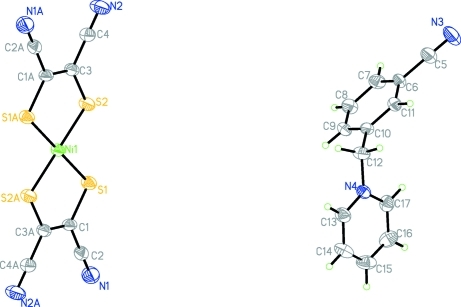
The molecular structure of (I), showing the atom-numbering scheme and displacement ellipsoids at the 30% probability level.

## supplementary crystallographic information

### Comment

Molecular solids based on transition metal dithiolene complexes have attracted intense interest in recent years, not only owing to the fundamental research of magnetic interactions and magneto-structural correlations but also to the development of new functional molecule-based materials (Robertson & Cronin, 2002), Much work has been performed in molecular solids based on *M*[dithiolene]_2_ complexes owing to their application as building blocks in molecular-based materials showing magnetic, superconducting, and optical properties (Nishijo *et al.*, 2000; Ni *et al.*, 2005). Herein we report the crystal structure of the title compound (I).

The molecular structure of (I) is illustrated in Fig. 1.The asymmetric unit is formed by with one half anion and one cation.The Ni ^II ^ion is coordinated by four sulfur atoms of two mnt^2-^ ligands, and exhibits square-planar coordination geometry. The crystal structure is stabilized by coulombic interactions. The bond lengths and angles are in good agreement with related compounds [Ni(mnt)_2_]^2- ^Table 1, (Ni *et al.*, 2004; Ren *et al.*, 2004).

### Experimental

Disodium maleonitriledithiolate (456 mg, 2.5 mmol) and nickel chloride hexahydrate (297 mg, 1.25 mmol) were mixed under stirring in water (20 mL) at room temperature. Subsequently, a solution of 1-(3-cyanobenzyl)pyridinium iodide (488 mg, 2.5 mmol) in methanol (10 mL) was added to the mixture, and the red precipitate that was immediately formed was filtered off, and washed with methanol. The crude product was recrystallized in acetone (20 mL) to give red block crystals. Anal. Calcd. for C_34_H_22_N_8_NiS_4_: C, 55.98; H, 3.04; N, 15.36%. Found: C, 56.00; H, 3.08; N, 15.33%.

### Refinement

The H atoms were placed to the bonded parent atoms in geometrically idealized positions (C—H = 0.93, or 0.97 Å) and refined as riding atoms, with *U*_iso_(H) = 1.2*U*_eq_(C).

### Crystal data

**Table:**

(C_13_H_11_N_2_)_2_[Ni(C_4_N_2_S_2_)_2_]	*Z* = 2
*M**_r_* = 729.57	*F*(000) = 748
Monoclinic, *P*2_1_/*c*	*D*_x_ = 1.433 Mg m^−^^3^
Hall symbol: -P 2ybc	Mo *K*α radiation, λ = 0.71070 Å
*a* = 11.633 (3) Å	θ = 3.0–25.4°
*b* = 8.709 (2) Å	µ = 0.86 mm^−^^1^
*c* = 16.692 (4) Å	*T* = 293 K
β = 91.278 (5)°	Block, red
*V* = 1690.7 (7) Å^3^	0.4 × 0.3 × 0.2 mm

### Data collection

**Table:**

Bruker SMART CCD area-detector diffractometer	3068 independent reflections
Radiation source: fine-focus sealed tube	2154 reflections with *I* > 2σ(*I*)
graphite	*R*_int_ = 0.092
phi and ω scans	θ_max_ = 25.4°, θ_min_ = 3.0°
Absorption correction: multi-scan (*SADABS*; Bruker, 2000)	*h* = −13→13
*T*_min_ = 0.702, *T*_max_ = 0.741	*k* = −9→10
13953 measured reflections	*l* = −20→20

### Refinement

**Table:**

Refinement on *F*^2^	Primary atom site location: structure-invariant direct methods
Least-squares matrix: full	Secondary atom site location: difference Fourier map
*R*[*F*^2^ > 2σ(*F*^2^)] = 0.076	Hydrogen site location: inferred from neighbouring sites
*wR*(*F*^2^) = 0.130	H-atom parameters constrained
*S* = 1.18	*w* = 1/[σ^2^(*F*_o_^2^) + (0.0263*P*)^2^ + 1.4994*P*] where *P* = (*F*_o_^2^ + 2*F*_c_^2^)/3
3068 reflections	(Δ/σ)_max_ < 0.001
214 parameters	Δρ_max_ = 0.32 e Å^−^^3^
0 restraints	Δρ_min_ = −0.31 e Å^−^^3^

### Special details

**Table:**

Geometry. All e.s.d.'s (except the e.s.d. in the dihedral angle between two l.s. planes) are estimated using the full covariance matrix. The cell e.s.d.'s are taken into account individually in the estimation of e.s.d.'s in distances, angles and torsion angles; correlations between e.s.d.'s in cell parameters are only used when they are defined by crystal symmetry. An approximate (isotropic) treatment of cell e.s.d.'s is used for estimating e.s.d.'s involving l.s. planes.
Refinement. Refinement of *F*^2^ against ALL reflections. The weighted *R*-factor *wR* and goodness of fit *S* are based on *F*^2^, conventional *R*-factors *R* are based on *F*, with *F* set to zero for negative *F*^2^. The threshold expression of *F*^2^ > σ(*F*^2^) is used only for calculating *R*-factors(gt) *etc*. and is not relevant to the choice of reflections for refinement. *R*-factors based on *F*^2^ are statistically about twice as large as those based on *F*, and *R*- factors based on ALL data will be even larger.

### Fractional atomic coordinates and isotropic or equivalent isotropic displacement parameters (Å^2^)

**Table:**

	*x*	*y*	*z*	*U*_iso_*/*U*_eq_	
Ni1	0.0000	0.0000	1.0000	0.0420 (3)	
S1	0.08308 (11)	0.02743 (15)	0.88570 (8)	0.0527 (4)	
S2	−0.06667 (12)	0.23125 (15)	0.98459 (8)	0.0543 (4)	
N1	0.2335 (5)	−0.2035 (6)	0.7340 (3)	0.0840 (16)	
N2	−0.2202 (5)	0.5312 (6)	1.0989 (3)	0.0818 (16)	
N3	0.5244 (5)	1.6046 (6)	0.9022 (4)	0.0906 (17)	
N4	0.7437 (3)	0.8647 (4)	0.9068 (2)	0.0440 (10)	
C1	0.1400 (4)	−0.1530 (6)	0.8694 (3)	0.0460 (12)	
C2	0.1931 (5)	−0.1824 (6)	0.7942 (3)	0.0557 (14)	
C3	−0.1341 (4)	0.2636 (6)	1.0743 (3)	0.0477 (13)	
C4	−0.1819 (4)	0.4130 (6)	1.0888 (3)	0.0522 (13)	
C5	0.5193 (5)	1.4752 (6)	0.8943 (3)	0.0636 (15)	
C6	0.5134 (4)	1.3103 (5)	0.8838 (3)	0.0476 (12)	
C7	0.4315 (4)	1.2492 (6)	0.8324 (3)	0.0560 (14)	
H11A	0.3794	1.3125	0.8053	0.067*	
C8	0.4284 (4)	1.0918 (6)	0.8219 (3)	0.0567 (14)	
H10A	0.3741	1.0489	0.7868	0.068*	
C9	0.5040 (4)	0.9987 (6)	0.8625 (3)	0.0502 (12)	
H9A	0.4994	0.8928	0.8559	0.060*	
C10	0.5869 (4)	1.0600 (5)	0.9132 (3)	0.0425 (12)	
C11	0.5915 (4)	1.2165 (5)	0.9241 (3)	0.0481 (13)	
H13A	0.6468	1.2592	0.9584	0.058*	
C12	0.6706 (4)	0.9595 (6)	0.9596 (3)	0.0561 (14)	
H7A	0.7197	1.0239	0.9932	0.067*	
H7B	0.6279	0.8921	0.9945	0.067*	
C13	0.7754 (4)	0.7251 (6)	0.9321 (3)	0.0587 (14)	
H4A	0.7510	0.6900	0.9815	0.070*	
C14	0.8426 (5)	0.6340 (7)	0.8868 (4)	0.0729 (17)	
H3A	0.8637	0.5367	0.9047	0.087*	
C15	0.8784 (5)	0.6860 (8)	0.8156 (4)	0.0707 (17)	
H2A	0.9225	0.6233	0.7833	0.085*	
C16	0.8498 (5)	0.8310 (8)	0.7906 (3)	0.0674 (16)	
H1A	0.8761	0.8686	0.7422	0.081*	
C17	0.7823 (4)	0.9196 (6)	0.8379 (3)	0.0568 (14)	
H6A	0.7632	1.0188	0.8220	0.068*	

### Atomic displacement parameters (Å^2^)

**Table:**

	*U*^11^	*U*^22^	*U*^33^	*U*^12^	*U*^13^	*U*^23^
Ni1	0.0495 (5)	0.0381 (5)	0.0383 (5)	0.0040 (4)	0.0040 (4)	−0.0014 (4)
S1	0.0691 (9)	0.0437 (8)	0.0457 (8)	0.0070 (7)	0.0132 (6)	0.0017 (6)
S2	0.0716 (9)	0.0436 (8)	0.0482 (8)	0.0121 (7)	0.0119 (7)	0.0030 (6)
N1	0.109 (4)	0.071 (4)	0.074 (4)	−0.005 (3)	0.038 (3)	−0.009 (3)
N2	0.107 (4)	0.057 (3)	0.082 (4)	0.028 (3)	0.022 (3)	−0.001 (3)
N3	0.113 (4)	0.041 (3)	0.117 (5)	0.000 (3)	−0.003 (4)	−0.004 (3)
N4	0.049 (2)	0.038 (2)	0.045 (3)	0.005 (2)	−0.004 (2)	0.003 (2)
C1	0.045 (3)	0.046 (3)	0.047 (3)	0.005 (2)	0.006 (2)	−0.012 (3)
C2	0.064 (3)	0.046 (3)	0.057 (4)	−0.003 (3)	0.013 (3)	−0.007 (3)
C3	0.050 (3)	0.042 (3)	0.052 (3)	0.006 (3)	0.003 (2)	−0.005 (3)
C4	0.057 (3)	0.051 (3)	0.048 (3)	0.008 (3)	0.004 (3)	−0.001 (3)
C5	0.070 (4)	0.046 (4)	0.074 (4)	0.005 (3)	−0.004 (3)	−0.001 (3)
C6	0.056 (3)	0.031 (3)	0.055 (3)	0.001 (2)	0.004 (3)	0.003 (2)
C7	0.054 (3)	0.054 (3)	0.060 (4)	0.014 (3)	−0.008 (3)	0.002 (3)
C8	0.054 (3)	0.055 (4)	0.060 (4)	0.003 (3)	−0.011 (3)	−0.009 (3)
C9	0.056 (3)	0.035 (3)	0.060 (3)	0.003 (3)	0.002 (3)	−0.005 (3)
C10	0.044 (3)	0.037 (3)	0.046 (3)	0.006 (2)	0.006 (2)	−0.002 (2)
C11	0.049 (3)	0.043 (3)	0.051 (3)	−0.003 (3)	0.000 (2)	−0.007 (2)
C12	0.069 (3)	0.052 (3)	0.048 (3)	0.018 (3)	0.008 (3)	0.000 (3)
C13	0.068 (4)	0.046 (3)	0.063 (4)	0.010 (3)	0.004 (3)	0.010 (3)
C14	0.092 (4)	0.049 (4)	0.078 (5)	0.021 (3)	−0.003 (4)	−0.010 (3)
C15	0.065 (4)	0.080 (5)	0.067 (4)	0.016 (3)	−0.006 (3)	−0.033 (4)
C16	0.061 (3)	0.093 (5)	0.049 (4)	0.008 (4)	0.005 (3)	0.003 (3)
C17	0.058 (3)	0.055 (3)	0.057 (4)	−0.002 (3)	−0.001 (3)	0.014 (3)

### Geometric parameters (Å, °)

**Table:**

Ni1—S1	2.1710 (13)	C7—H11A	0.9300
Ni1—S1^i^	2.1710 (13)	C8—C9	1.365 (6)
Ni1—S2	2.1713 (14)	C8—H10A	0.9300
Ni1—S2^i^	2.1713 (13)	C9—C10	1.377 (6)
S1—C1	1.729 (5)	C9—H9A	0.9300
S2—C3	1.729 (5)	C10—C11	1.376 (6)
N1—C2	1.134 (6)	C10—C12	1.510 (6)
N2—C4	1.135 (6)	C11—H13A	0.9300
N3—C5	1.136 (6)	C12—H7A	0.9700
N4—C17	1.332 (6)	C12—H7B	0.9700
N4—C13	1.336 (6)	C13—C14	1.356 (7)
N4—C12	1.489 (6)	C13—H4A	0.9300
C1—C3^i^	1.348 (6)	C14—C15	1.347 (8)
C1—C2	1.435 (7)	C14—H3A	0.9300
C3—C1^i^	1.348 (6)	C15—C16	1.368 (8)
C3—C4	1.438 (7)	C15—H2A	0.9300
C5—C6	1.449 (7)	C16—C17	1.365 (7)
C6—C7	1.374 (7)	C16—H1A	0.9300
C6—C11	1.385 (6)	C17—H6A	0.9300
C7—C8	1.382 (7)		
			
S1—Ni1—S1^i^	180.0	C8—C9—H9A	119.7
S1—Ni1—S2	87.64 (5)	C10—C9—H9A	119.7
S1^i^—Ni1—S2	92.36 (5)	C11—C10—C9	119.3 (5)
S1—Ni1—S2^i^	92.36 (5)	C11—C10—C12	119.0 (5)
S1^i^—Ni1—S2^i^	87.64 (5)	C9—C10—C12	121.7 (4)
S2—Ni1—S2^i^	180.0	C10—C11—C6	119.8 (5)
C1—S1—Ni1	102.59 (18)	C10—C11—H13A	120.1
C3—S2—Ni1	102.48 (17)	C6—C11—H13A	120.1
C17—N4—C13	120.3 (4)	N4—C12—C10	112.8 (4)
C17—N4—C12	121.3 (4)	N4—C12—H7A	109.0
C13—N4—C12	118.3 (4)	C10—C12—H7A	109.0
C3^i^—C1—C2	120.8 (4)	N4—C12—H7B	109.0
C3^i^—C1—S1	120.9 (4)	C10—C12—H7B	109.0
C2—C1—S1	118.3 (4)	H7A—C12—H7B	107.8
N1—C2—C1	178.5 (6)	N4—C13—C14	120.9 (5)
C1^i^—C3—C4	120.2 (4)	N4—C13—H4A	119.5
C1^i^—C3—S2	121.2 (4)	C14—C13—H4A	119.5
C4—C3—S2	118.5 (4)	C15—C14—C13	119.3 (6)
N2—C4—C3	178.9 (6)	C15—C14—H3A	120.4
N3—C5—C6	179.6 (8)	C13—C14—H3A	120.4
C7—C6—C11	120.9 (5)	C14—C15—C16	120.1 (6)
C7—C6—C5	119.4 (5)	C14—C15—H2A	120.0
C11—C6—C5	119.8 (5)	C16—C15—H2A	120.0
C6—C7—C8	118.6 (5)	C17—C16—C15	119.0 (6)
C6—C7—H11A	120.7	C17—C16—H1A	120.5
C8—C7—H11A	120.7	C15—C16—H1A	120.5
C9—C8—C7	120.7 (5)	N4—C17—C16	120.4 (5)
C9—C8—H10A	119.6	N4—C17—H6A	119.8
C7—C8—H10A	119.6	C16—C17—H6A	119.8
C8—C9—C10	120.6 (5)		

Symmetry codes: (i) −*x*, −*y*, −*z*+2.
